# Detection of Eight Respiratory Bacterial Pathogens Based on Multiplex Real-Time PCR with Fluorescence Melting Curve Analysis

**DOI:** 10.1155/2020/2697230

**Published:** 2020-02-26

**Authors:** Liuyang Hu, Bing Han, Qin Tong, Hui xiao, Donglin Cao

**Affiliations:** ^1^Department of Laboratory Medicine, Guangdong Second Provincial General Hospital, Guangzhou 510317, China; ^2^The Second School of Clinical Medicine, Southern Medical University, Guangzhou 510515, China; ^3^Shenzhen Longhua District Central Hospital, Shenzhen 518110, China; ^4^Guangzhou Baochuang Biotechnology Co., Ltd., Guangzhou 510336, China

## Abstract

*Background and Objective*. *Acinetobacter baumannii*, *Escherichia coli*, *Klebsiella pneumoniae*, *Streptococcus pneumoniae*, *Haemophilus influenzae*, *Staphylococcus aureus*, *Pseudomonas aeruginosa*, and *Mycobacterium tuberculosis* are primary respiratory bacterial pathogens contributing to morbidity and mortality in developing countries. This study evaluated the diagnostic performance of multiplex real-time PCR with fluorescence melting curve analysis (MCA) assay, which was used to detect eight respiratory bacterial pathogens simultaneously.

**Methods:**

A total of 157 sputum specimens were examined by multiplex real-time with fluorescence MCA, and the results were compared with the conventional culture method.

**Results:**

Multiplex real-time PCR with fluorescence MCA specifically detected and differentiated eight respiratory bacterial pathogens by different melting curve peaks for each amplification product within 2 hours and exhibited high repeatability. The limit of detection ranged from 64 to 10^2^ CFU/mL in the multiplex PCR system. Multiplex real-time PCR with fluorescence MCA showed a sensitivity greater than 80% and a 100% specificity for each pathogen. The kappa correlation of eight bacteria ranged from 0.89 to 1.00, and the coefficient of variation ranged from 0.05% to 0.80%.

**Conclusions:**

Multiplex real-time PCR with fluorescence MCA assay is a sensitive, specific, high-throughput, and cost-effective method to detect multiple bacterial pathogens simultaneously.

## 1. Introduction

Lower respiratory tract infections are among the leading causes of hospitalization and death around the world [1–4]. The causative agents of lower respiratory tract infections are diverse [5], while respiratory bacterial pathogens remain to be the primary cause [6–9]. *S. aureus*, *E. coli*, and *K. pneumoniae* commonly cause lower respiratory tract infections in children [7]. In addition, bacteria such as *K. pneumoniae*, *P. aeruginosa*, and *S. pneumoniae* are also widely recognized as major pathogens of community-acquired pneumonia [10]. Currently, traditional detection methods for respiratory pathogens largely include the use of microscopic examination, bacterial culture, and immunological examination. These methods are complicated, time-consuming, technically difficult, and low sensitivity. Most importantly, these methods are not able to detect multiple pathogens in a single specimen. However, coinfection of respiratory pathogens is a common occurrence, especially in less developed countries such as China [8, 11]. As a result, more studies are aimed at developing strategies for the rapid and simultaneous identification of more than one pathogen by developing multiple analytic platforms. Although Luminex assays and TaqMan array cards can detect a variety of bacterial and viral pathogens simultaneously [12–14], the high cost of these methods limits their widespread application. In light of this, molecular diagnostic methods, especially real-time PCR, have surfaced as the most promising approach for detecting respiratory pathogens owing to their rapidity, sensitivity, and specificity. The two main types of real-time PCR are fluorescent probe-based assays and fluorescent dye-based assays. Numerous pathogen platforms based on TaqMan probes have been developed in recent years [15–17], but TaqMan probes are costly and complex to synthesize and have high potential false negative rates [18–20]. Meanwhile, real-time PCR applies simple DNA binding dyes as the signal reporter to detect the total amount of DNA through changes in fluorescence intensity [21]. Compared to TaqMan probes, fluorescent dyes have the merits of generalizability and cost-effectiveness. Nevertheless, current real-time PCR methods can only detect two or three pathogens simultaneously in a single reaction [22, 23] because of the limited recognition capabilities of the instrument. Consequently, fluorescence melting curve analysis (MCA) based on dyes was developed to overcome these limitations [24]. SYBR Green I is the most commonly used dye for real-time multiplex PCR [25]. However, the performance of MCA using SYBR Green I is problematic as the melting temperature of the dye can be easily affected by the dye concentration [26] and DNA concentration [27]. These shortcomings limit the application of SYBR Green I for melting curve analysis based on multiplex PCR [28]. In contrast, another dye, EvaGreen, has been found to be more sensitive and stable than SYBR Green I, owing to its ability to insert into each single-nucleotide saturated DNA helix [29]. At present, the melting curve analyses based on EvaGreen dye can simultaneously detect various plant viruses [24, 30] and allow sensitive, specific, and low-cost detection.

In order to realize the simultaneous detection of respiratory bacterial coinfections and improve the accuracy of detecting respiratory bacterial pathogens, the current study aims to establish an assay that combines multiplex real-time PCR techniques with EvaGreen MCA to detect eight common respiratory bacteria simultaneously.

## 2. Materials and Method

### 2.1. Bacterial Strains

Standard strains of *A. baumannii* (ATCC49619), *E. coli* (ATCC8739), *K. pneumoniae* (ATCC13883), *S. pneumoniae* (ATCC49619), *H. influenzae* (ATCC49766), *S. aureus* (ATCC6538), *P. aeruginosa* (ATCC0927), and *M. tuberculosis* were purchased from the Guangdong Culture Collection Center. Lyophilized bacteria from the aforementioned standard strains were inoculated into the corresponding medium and incubated at 37°C with 5% CO_2_ in air for 24 hours, and single colonies of the bacteria were obtained and stored in physiological saline.

### 2.2. Clinical Samples

Sputum samples from 157 cases were analyzed using multiplex real-time PCR with EvaGreen melting curve analysis, and the results were compared with the conventional culture method. All specimens were collected from patients hospitalized in Respiratory Department of the Guangdong Second Provincial General Hospital from November 2018 to June 2019, presenting with symptoms of cough and expectoration.

### 2.3. Sample Preparation

After adding equal amounts of 4% sodium hydroxide for 10 min, the sputum samples were taken in 1.5 mL centrifuge tubes, centrifuged at 15000 rpm/min for 5 min, and the supernatant was discarded. Next, after the addition of 1 mL of sterile physiological saline, the pellet was resuspended by shaking and centrifuged at 15000 rpm/min for 5 min, and the supernatant was discarded as much as possible and finally added with 1 mL of sterile physiological saline. The pretreated sputum samples were stored in tubes at −80°C. Genomic DNA content was extracted from the sputum samples using Magen HiPure Bacterial DNA kits according to the manufacturer's instructions.

### 2.4. Primers Design

The primers were designed so as to prepare amplicons having melting temperatures (*T*_*m*_s) ranging from 76°C to 92°C, with the difference between two consecutive peaks being more than 1°C. We employed the amplicon *T*_*m*_ as parameter to estimate whether the selected bacteria gene would produce a desired *T*_*m*_ of amplicon. The primers were designed using the Primer Premier 5.0 based on the selected areas which can produce amplicons with the expected *T*_*m*_. All primers were synthesized by Invitrogen (Karlsruhe, Germany). Eight selected bacterial primers (shown in Table 1) were added to the multiplex PCR system sequentially to determine their actual *T*_*m*_s and whether nonspecific primer amplicon would occur in the presence of other oligonucleotide primers.

### 2.5. PCR System and Program

Singleplex PCR was performed in a total volume of 25 *μ*L, which included 2.5 *μ*L DNA template, 0.6 *μ*M forward and reverse primers, 0.2 *μ*L of Taq DNA polymerase, 2 *μ*L dNTP mixture, 1 *μ*L of EvaGreen, 15.6 *μ*L of deionized water, and 2.5 *μ*L of 10 × PCR buffer. Meanwhile, multiplex real-time PCR was performed in a total volume of 25 *μ*L, which included 0.4 *μ*L of Taq DNA polymerase, 13.4 *μ*L of deionized water, and 0.2 *μ*L of each forward and reverse primer. The composition of the other reactions was determined by singleplex PCR. PCR amplifications were performed using the Roche LightCycler 480 system. The cycling condition included preincubation at 95°C for 3 minutes and the following amplification program for a total of 38 cycles: denaturation, 95°C, for 10 s; annealing, 60°C for 40 s; fluorescence collection, 72°C to 95°C; and cooling cycle, 50°C for 30 s. The melting temperature (*T*_*m*_) was calculated by plotting the negative derivative of fluorescence over temperature versus temperature (−d*F*/d*T* versus *T*). The *T*_*m*_ value was defined as the peak of the curve. Melting curves were used to determine the specific PCR products, which were further confirmed using conventional gel electrophoresis, and the PCR products which were inconsistent with the clinical results were sent for sequencing.

## 3. Result

### 3.1. Specificity and Sensitivity

**Figure 1:**
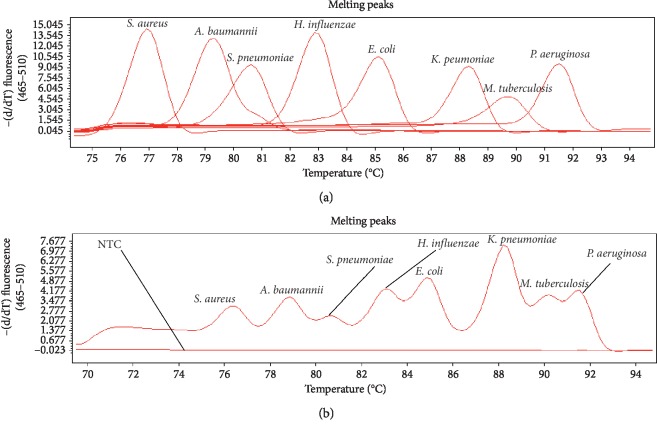
The specificity of EvaGreen multiplex real-time PCR protocol for the eight bacteria. (a) Results obtained for each bacterium when tested by multiplex real-time PCR protocol. (b) Each distinct peak corresponded to a specific bacterial standard strain DNA amplified by multiplex real-time PCR protocol, where nontemplate negative controls (NTC) did not show any peaks.

Initially, we evaluated the multiplex assay to determine whether the eight primers would amplify well in multiplex real-time PCR and whether nonspecific amplification or interference might occur. The results indicated that our system exhibited good specificity. The detailed process was as follows: each of the individual bacterium was tested under multiplex real-time PCR conditions, and eight bacteria were tested simultaneously, with sterile purified water serving as the negative control. Eight different *T*_*m*_ peaks were generated using PCR *T*_*m*_ analysis based on single real-time PCR. The mean *T*_*m*_ values for the bacterial strains of *S. aureus*, *A. baumannii*, *S. pneumoniae*, *H. influenzae*, *E. coli*, *K. pneumoniae*, *M. tuberculosis*, and *P. aeruginosa*, obtained by testing aliquots of individual bacterium in single PCR, were 76.40, 79.46, 80.50, 82.29, 84.49, 87.79, 89, and 91.01°C, respectively. The *T*_*m*_ peaks of the aforementioned bacteria were also observed in the multiplex PCR system for eight bacteria individually (Table 1; Figure 1(a)). In addition, multiplex real-time PCR was applied to detect the eight bacteria together, and the melting peaks of the amplicons were different such that multiple pathogens could be identified on a single graph unambiguously. The spacing between peaks for eight bacteria is shown in Figure 1(b). Meanwhile, nontarget bacteria and sterile purified water were used as templates for multiplex real-time PCR, and the specific *T*_*m*_ peaks of the eight bacterial templates were not seen except for the low *T*_*m*_ peaks that produced primer dimers. The primer dimers could be distinguished from the expected product (Figure 1(b)). Furthermore, to confirm target-specific amplification, the amplicons were analyzed with 3% agarose gel electrophoresis, which indicated that the assay based on multiplex real-time PCR with EvaGreen MCA was compatible with the use of eight primers and specific to the eight respiratory bacteria.

**Figure 2:**
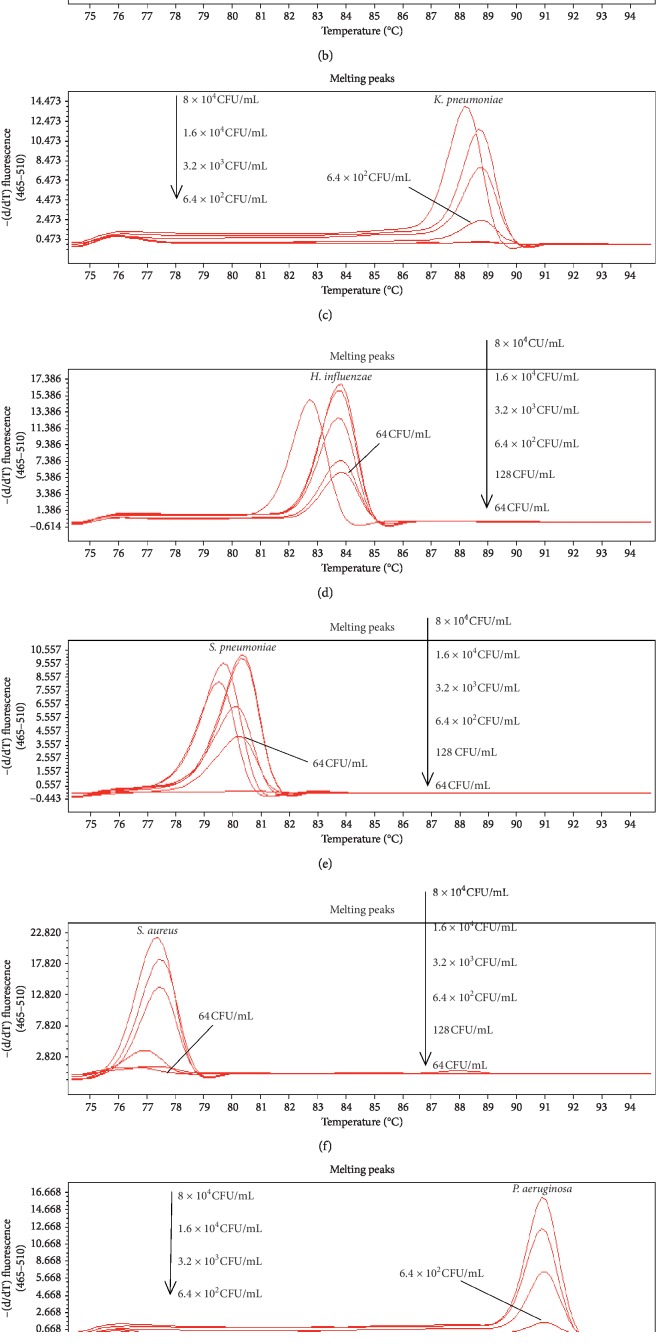
Sensitivity of multiplex real-time PCR protocol for detection of single bacterium.

**Figure 3:**
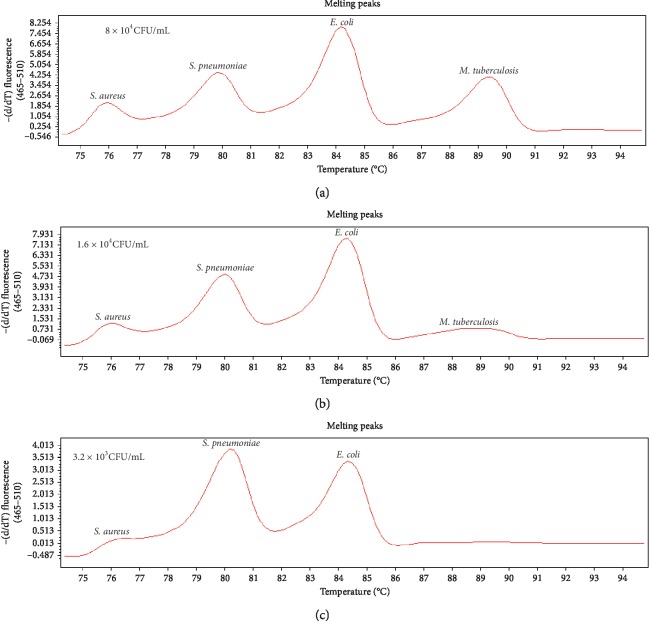
The sensitivity of simultaneous detection of *S. aureus*, *S. pneumoniae*, *E. coli*, and *M. tuberculosis*; the specific *T*_*m*_ peak of *M. tuberculosis* disappears when each bacterial concentration drops to 3.2 × 10^3^ CFU/mL.

The limit of detection (LOD) of bacterial DNA was evaluated by observing the specific *T*_*m*_ peaks of each bacterium using 5-fold serial dilutions of DNA: 64, 128, 6.4 × 10^2^, 3.2 × 10^3^, 1.6 × 10^4^, 8 × 10^4^, 4 × 10^5^, 2 × 10^6^, 10^7^ CFU/mL. The sensitivity of single real-time PCR detection was 128 CFU/mL for *P. aeruginosa* and 64 CFU/mL for the other bacteria. Single bacterium sensitivity of multiplex PCR system was as follows: 64 CFU/mL for *S. aureus*, *S. pneumoniae*, and *H. influenzae*; 128 CFU/mL for *A. baumannii*; and 6.4 × 10^2^ CFU/mL for *E. coli*, *K. pneumoniae*, *P. aeruginosa*, and *M. tuberculosis* (Figure 2). Single bacterium sensitivity of multiplex PCR system was 5–100 times lower than single real-time PCR. Considering the fact that current day respiratory infections are a mix of 2–4 bacteria, we tested the sensitivity of quadruple PCR for any four combinations of the eight bacteria. The sensitivity of simultaneous detection of four bacteria was 5–50 times lower than detection of single bacterium by multiplex PCR. When the bacterial concentration was as low as 3.2 × 10^3^ to 1.6 × 10^4^ CFU/mL, some of the specific *T*_*m*_ peaks disappeared. The current study investigated the sensitivity of simultaneous detection of *S. aureus*, *S. pneumoniae*, *E. coli*, and *M. tuberculosis*, while the results of other bacterial combinations were not shown. When the bacterial DNA concentration diminished to 3.2 × 10^3^ CFU/mL, the specific *T*_*m*_ peak of *M. tuberculosis* disappeared (Figure 3).

### 3.2. Standard Curves of Multiplex PCR System

**Figure 4:**
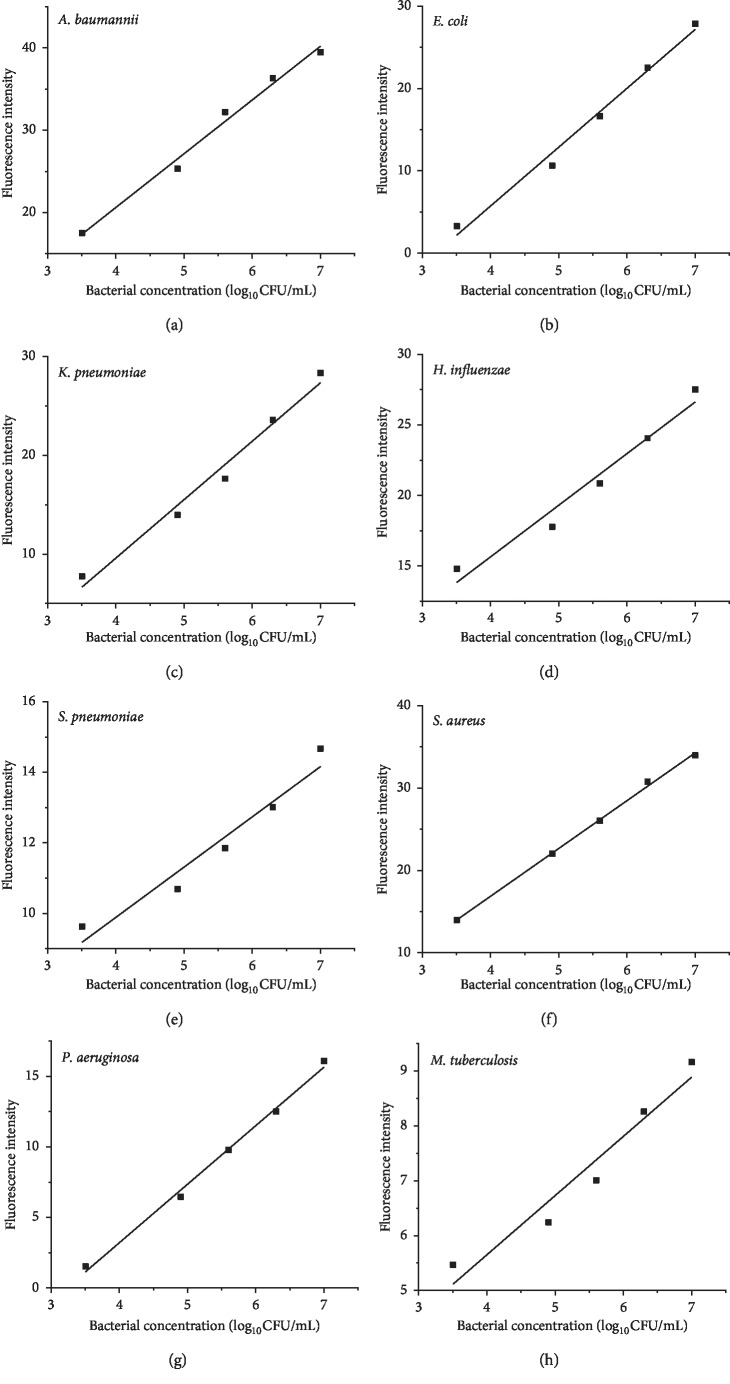
(a)–(h) Standard curves were obtained by plotting the fluorescence intensity (*y*-axis) values against the log_10_ of the bacterial concentration (*x*-axis) of the eight respective standard bacterial strains. The curve equations and R^2^ are as follows: *y* = 6.53*x* − 5.52, *R*^2^ = 0.988 for *A. baumannii* (a); *y* = 7.15*x* − 22.87, *R*^2^ = 0.987 for *E. coli* (b); *y* = 5.92*x* − 14.08, *R*^2^ = 0.980 for *K. pneumoniae* (c); *y* = 3.66*x* + 1.02, *R*^2^ = 0.965 for *H. influenzae* (d); *y* = 1.42*x* + 4.19, R^2^ = 0.947 for *S. pneumoniae* (e); *y* = 5.81*x* − 6.37, *R*^2^ = 0.998 for *S. aureus* (f); *y* = 4.15x − 13.39, *R*^2^ = 0.995 for *P. aeruginosa* (g); *y* = 1.08*x* + 1.34, *R*^2^ = 0.943 for *M. tuberculosis* (h).

Standard curves of multiplex PCR system for each bacterium were obtained by plotting the fluorescence intensity (−d*F*/d*T*) (*y*-axis) values against the log_10_ of the bacterial concentration (*x*-axis) of the eight respective standard bacterial strains. The dynamic range of the assay encompassed at least five orders of magnitude, with a strong linear relationship between the fluorescence intensity (−d*F*/d*T*) values and the log_10_ value of the bacterial concentration ranging from 10^3^ to 10^7^ CFU/mL (Figure 4). The correlation coefficients (*R*^2^) of the standard curve for eight bacteria in multiplex PCR system were all beyond 0.94.

### 3.3. Repeatability of Multiplex Real-Time PCR with EvaGreen Melting Curve Analysis

The repeatability of EvaGreen dye dissolution curve was evaluated by multiplex real-time PCR detection of triplicate 1.6 × 10^4^, 8 × 10^4^, 4 × 10^5^ CFU/mL of eight bacterial standard strains' DNA mixed equally. Subsequently, standard deviation (SD) and coefficient of variation (CV) between measurements were calculated. SD of each bacterium was less than 1°C, while the intra-assay CV ranged from 0.05% to 0.6%, and the inter-assay CV ranged from 0.2% to 0.8%. The data suggested that this assay had good reproducibility and also revealed that two closely spaced *T*_*m*_ peaks were able to separate well.

### 3.4. Clinical Sample Testing and Statistical Analysis

The EvaGreen MCA assay was employed to analyze 157 samples of sputum specimens, and the results were compared with the conventional culture method. Conventional culture method revealed that there were 30 cases of *A. baumannii*, 12 cases of *E. coli*, 18 cases of *K. pneumoniae*, 5 cases of *H. influenzae*, 2 cases of *S. pneumoniae*, 13 cases of *S. aureus*, 17 cases of *P. aeruginosa*, and 10 cases of *M. tuberculosis*, with semiquantitative culture being more than ++. Meanwhile, 50 cases were negative samples. 73 samples out of 107 culture positive samples were identified as 2–4 pathogens, and 50 culture negative samples were confirmed as negative by EvaGreen MCA assay. 64 samples out of the 73 multiple pathogens cases showed 2–4 melting curve peaks, which were considered to have 2–4 pathogens, but only one of the curve peaks exhibited strong fluorescence signal, and the other curve peaks showed weak fluorescence signals. Typically, pathogenic bacterial concentration ≥10^5^ CFU/mL is considered as bacterial infection [31, 32]. According to the standard curve, the multiple pathogens that had weak fluorescence signals in these 64 samples of 73 cases were regarded as colonizing pathogens, and only the bacterium with strong fluorescence signal was identified as infection. Therefore, the 64 samples with one strong fluorescence signal only were considered as monoinfections. However, the remaining 9 samples showed 2-3 melting curve peaks, which all exhibited strong fluorescence signals. Consequently, the 9 samples were identified as 2-3 bacterial coinfections, which are shown in Table 2. Overall, according to the results of 157 clinical samples, the sensitivity of *A. baumannii*, *E. coli*, *K. pneumoniae*, *H. influenzae*, *S. pneumoniae*, *P. aeruginosa*, *S. aureus*, and *M. tuberculosis* was 100%, 91.7%, 100%, 80%, 100%, 100%, 100%, and 90%, respectively. The specificity was 100% for all targets. The consistency between EvaGreen MCA assay and conventional culture method was 98%, and the kappa value ranged from 0.89 to 1.00 (Table 3).

## 4. Discussion

In the current study, we successfully developed a multiplex real-time PCR with MCA fluorescent dye to identify and distinguish eight respiratory bacterial pathogens simultaneously in a single reaction tube. Our evidence revealed that all target bacterial DNA could be identified accurately and cross-reactivity was not present. The LOD for individual bacterium in the multiplex real-time PCR system ranged from 64 to 10^2^ CFU/mL. In addition, we discovered that the dissolution curve detection method could accurately detect multiple pathogens in a single reaction tube. We assessed 157 clinical samples, for which the sensitivity was greater than 80% and the specificity was 100%, and the kappa correlation values of all pathogens ranged from 0.89 to 1.00. The results indicated that using multiplex PCR with EvaGreen MCA for the detection of multiple pathogens simultaneous is sensitive, specific, high-throughput, and low-cost method.

Numerous researchers have devoted their efforts in developing molecular methods to solve the problem of multiple detection in a reaction [33, 34]. Currently, there are various studies focusing on the multiplex real-time PCR dissolution curve method [35, 36], because the PCR dissolution curve method based on EvaGreen dye is rapid, efficient, sensitive, specific, and low cost [24, 30]. The noteworthy characteristic of this methodology is the implementation of well-designed primers to make each detection target have different *T*_*m*_ values. However, it is not feasible to predict accurate *T*_*m*_ values using algorithms under actual PCR conditions as several factors may affect the absolute position of the DNA dissolution curve [37]. In the current study, we designed eight specific amplicons—one for each pathogen here investigated—with different *T*_*m*_ values. Pathogen-specific *T*_*m*_ values differed from each other by at least 1°C. Studies have been performed to describe changes in genotypes other than 1°C [38, 39]. We discovered that, although the *T*_*m*_ value of the dissolution curve did change, the eight bacteria were clearly distinguishable within the interval of 1°C. In addition, we found that the eight pairs of primers may exhibit primer dimerization; however, the primer dimer has no specific *T*_*m*_ peak, so it does not affect the overall results. We also revealed that the sensitivity of single bacterium in multiplex PCR was 5–100 times lower than that of single real-time PCR, and the sensitivity of four bacteria using quadruple PCR system was 5–50 times lower than that of single bacterium using multiplex PCR. This phenomenon can be explained by the fact that combination of primers in a single reaction may cause specific primers to be less available than required. Moreover, the presence of multiple targets in one reaction may also lead to competition between enzymes and nucleotides.

Quantification of the target pathogens is the key to distinguish between bacterial infection and bacterial colonization. In the current study, analysis results revealed a good correlation (R^2^) and a linear relationship between the fluorescence values and log_10_ of the bacterial concentration ranging from 10^3^ to 10^7^ CFU/mL in a multiplex real-time PCR system for one bacterium; we can approximately judge the concentration of single bacterium. Furthermore, it was found that the linear relationship between the fluorescence values and log_10_ of the bacterial concentration was weakened in a multitarget reaction, which can be attributed to the competition between EvaGreen dyes. Thus, the quantification of multiple pathogens in one reaction is not an accurate method. Herein lies the distinct advantage of EvaGreen MCA assay, that this method can detect mixed pathogens in a single sample. EvaGreen MCA assay can diagnose pathogens quickly and accurately in early stage, which is helpful for the rapid administration of appropriate treatment and, consequently, resolution of symptoms. We employed the EvaGreen assay to evaluate 157 cases of clinical sputum samples, which identified 73 cases as multiple pathogens. Amongst the identified 73 cases, 9 cases were defined as cases of bacterial coinfection and 64 samples were considered as monoinfections. However, the conventional culture method defined these 9 coinfected cases as single bacterial infection. After reviewing the history of the abovementioned 9 patients, it was found that these patients were hospitalized for long durations, and the treatment time for infection was more than one month, which is characteristic of difficult mixed infection treatment. Yet, the gold standard for clinical diagnosis remains to be bacterial culture which picks up single colonies of dominant bacteria. Consequently, the most predominant bacterial species are generally identified and misdiagnosis of mono infections may occur. In our study, the reason of the discrepant results about these 9 samples between the EvaGreen MCA and conventional culture method may be due to the minor single colonies of the samples were ignored or not cultured. Multiplex real-time PCR based on EvaGreen MCA allows us to directly detect the DNA of sputum samples, making it easier to identify multiple bacterial infections. EvaGreen MCA is a rapid and accurate assay; the total time of sample preparation, DNA extraction, PCR, and melting curve analysis is less than 2 hours. The drawback of EvaGreen MCA is inaccurate quantification of multiple pathogens in one reaction, so clinicians must make a comprehensive judgment based on the source of samples and disease conditions to distinguish colonization and infection. In order to overcome this shortcoming, dissolution curve interpretation software will be developed in the future based on the detection of a large number of clinical samples.

## 5. Conclusions

In summary, the current study highlighted EvaGreen MCA as a sensitive, specific, rapid, cost-effective, and high-throughput method for the detection and differentiation of eight clinically common respiratory bacteria. We hope our findings will serve as an effective platform for detection of respiratory bacterial coinfection in the future.

## Figures and Tables

**Table 1:** Primers designed for eight bacteria pathogens used for single and multiplex real-time PCR.

Primer	Gene	Sequence (5′-3′)	Amplicon size (bp)	Calculated *T*_*m*_^a^	Intra-assay *T*_*m*_^b^	Inter-assay *T*_*m*_^c^
*A. baumanni*i-F	iucD	GGCTGGACATCATCAACTGC	193	79.46	78.89 ± 0.03	79.44 ± 0.25
*A. baumannii*-R		GTCGGCCTGATCTCGTATGA				
*E. coli*-F	uidA	CGACTGGGCAGATGAACATG	225	84.49	84.48 ± 0.09	85.08 ± 0.33
*E. coli*-R		TACTCCACATCACCACGCTT				
*K. pneumoniae*-F	yfkN	TACACAATCGCCCGTTGAAC	223	87.79	87.23 ± 0.47	88.02 ± 0.68
*K. pneumoniae*-R		CCCGGTTAGATCCATGGTGA				
*H. influenzae*-F	atoE	CTGGTGTTGCGGCTAAAAGT	168	82.29	82.73 ± 0.21	83.21 ± 0.55
*H. influenzae*-R		TCATTAACTGGGGCTTCGGT				
*S. pneumoniae*-F	lytA	GCACACTCAACTGGGAATCC	110	80.50	80.10 ± 0.13	80.49 ± 0.41
*S. pneumoniae*-R		ATGCAACCGTTCCCAACAAT				
*S. aureus*-F	cap5F	AGTCACGTCTCGATCGAACA	175	76.40	76.33 ± 0.25	77.02 ± 0.16
*S. aureus*-R		GAAACTTGACCACGATCCGG				
*P. aeruginosa*-F	hpmA	AGAAGACCGTAAGCCAGACC	244	91.01	90.92 ± 0.05	90.94 ± 0.14
*P. aeruginosa*-R		CTACTGGCACCCACTCCTG				
*M. tuberculosis*-F	IS6110	GTCTACTTGGTGTTGGCTGC	108	89	89.52 ± 0.06	89.65 ± 0.03
*M. tuberculosis*-R		TCAGCTCAGCGGATTCTTCG				

^a^Obtained by using Primer Premier 5.0. ^b,c^Obtained by EvaGreen MAC of multiplex real-time PCR, mean ± SD, corresponding to nine replicates.

**Table 2:** Coinfected samples of EvaGreen MCA assay compared with the conventional culture.

Sample number	Conventional culture method	EvaGreen MCA
1	*A. baumannii*	*A. baumannii*, *E. coli*
2	*A. baumannii*	*A. baumannii*, *K. pneumoniae*
3	*E. coli*	*E. coli*, *P. aeruginosa*
4	*E. coli*	*E. coli*, *A. baumannii*
5	*P. aeruginosa*	*P. aeruginosa*, *K. pneumoniae*
6	*P. aeruginosa*	*P. aeruginosa*, *A. baumannii*
7	*P. aeruginosa*	*P. aeruginosa*, *A. baumannii*, *K. pneumoniae*
8	*K. pneumoniae*	*K. pneumoniae*, *E. coli*
9	*K. pneumoniae*	*K. pneumoniae*, *A. baumannii*

**Table 3:** Comparison of the results between EvaGreen MCA and conventional culture method.

Bacterial pathogens	EvaGreen MCA	Conventional culture method		Sensitivity (%)	Specificity (%)	Agreement (%)	Kappa
		Positive	Negative				
*A. baumannii*	Positive	30	0	100	100	100	1.00
	Negative	0	127				
*E. coli*	Positive	11	0	91.7	100	99.4	0.95
	Negative	1	145				
*K. pneumoniae*	Positive	18	0	100	100	100	1.00
	Negative	0	139				
*H. influenzae*	Positive	4	0	80	100	99.4	0.89
	Negative	1	152				
*S. pneumoniae*	Positive	2	0	100	100	100	1.00
	Negative	0	155				
*S. aureus*	Positive	13	0	100	100	100	1.00
	Negative	0	144				
*P. aeruginosa*	Positive	17	0	100	100	100	1.00
	Negative	0	140				
*M. tuberculosis*	Positive	9	0	90	100	99.4	0.94
	Negative	1	147				

## Data Availability

The data used to support the findings of this study are available from the corresponding author upon request.
